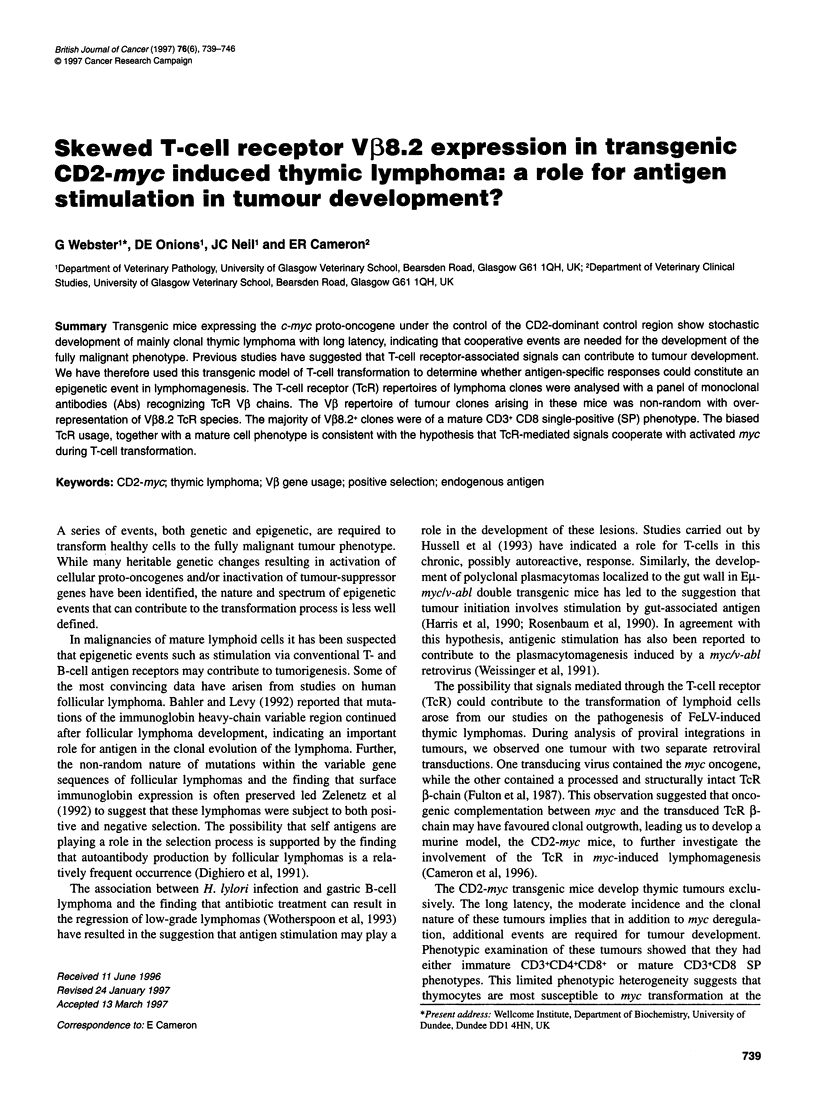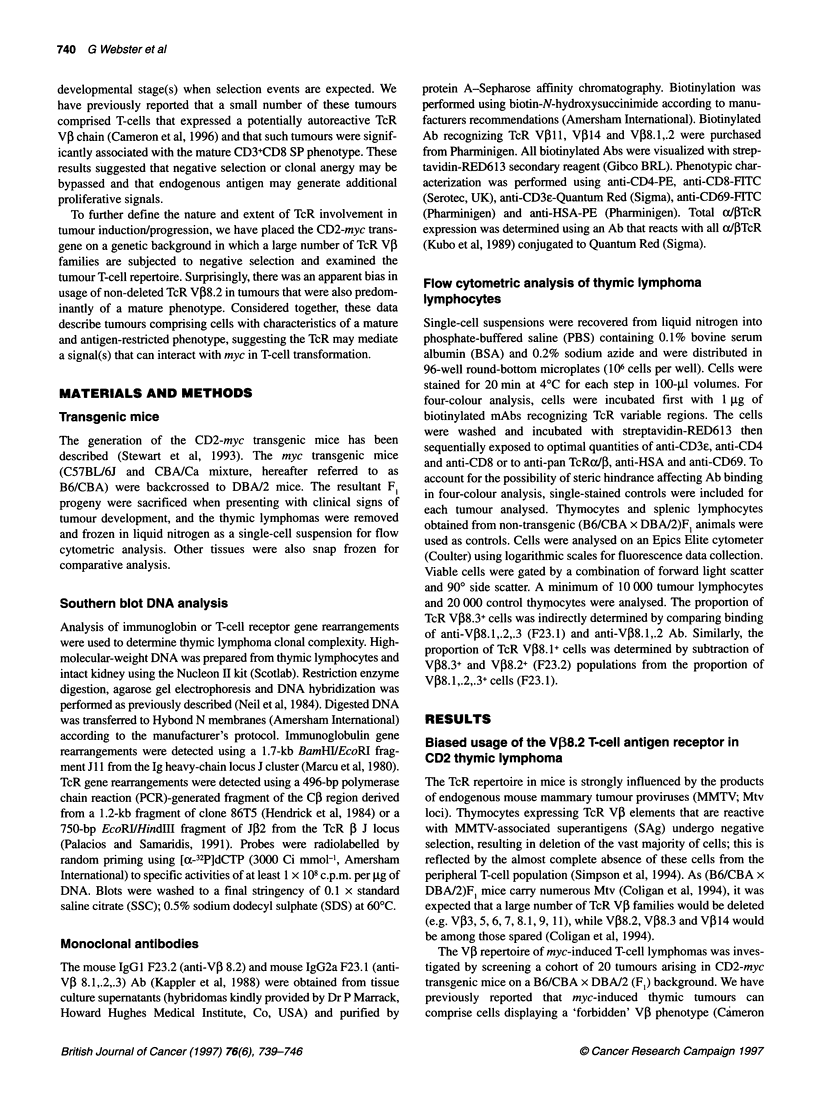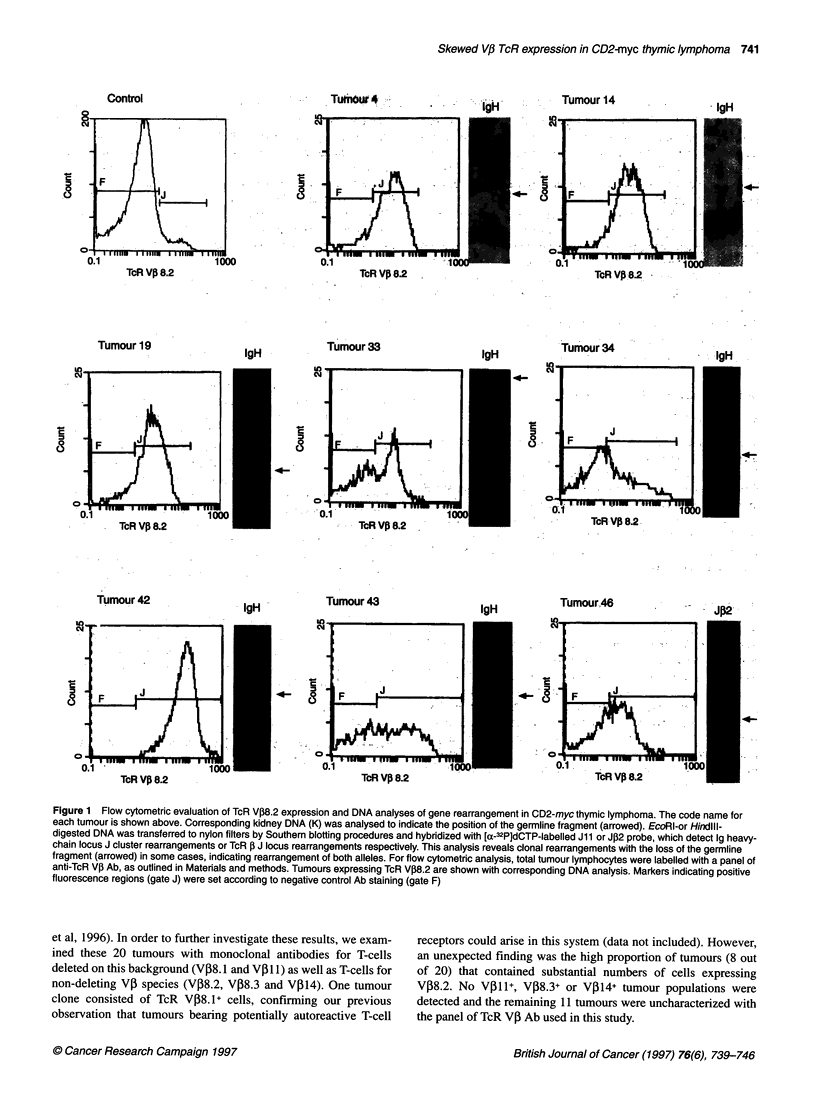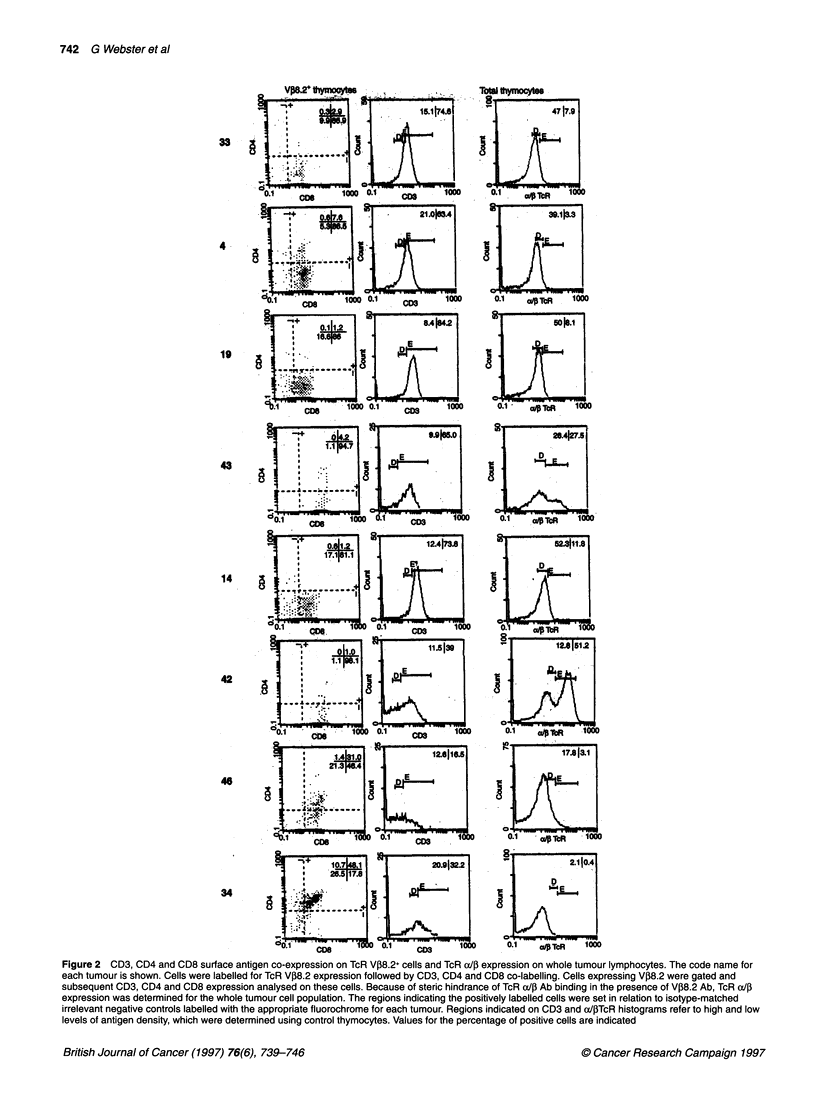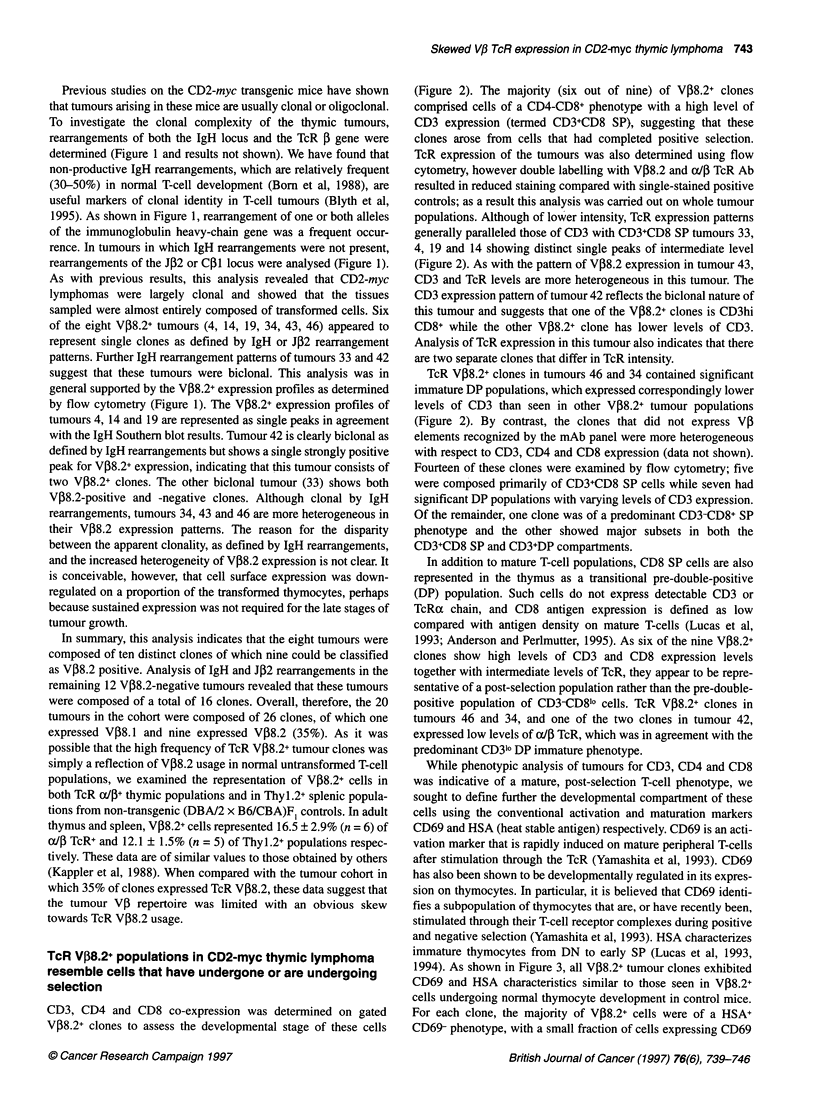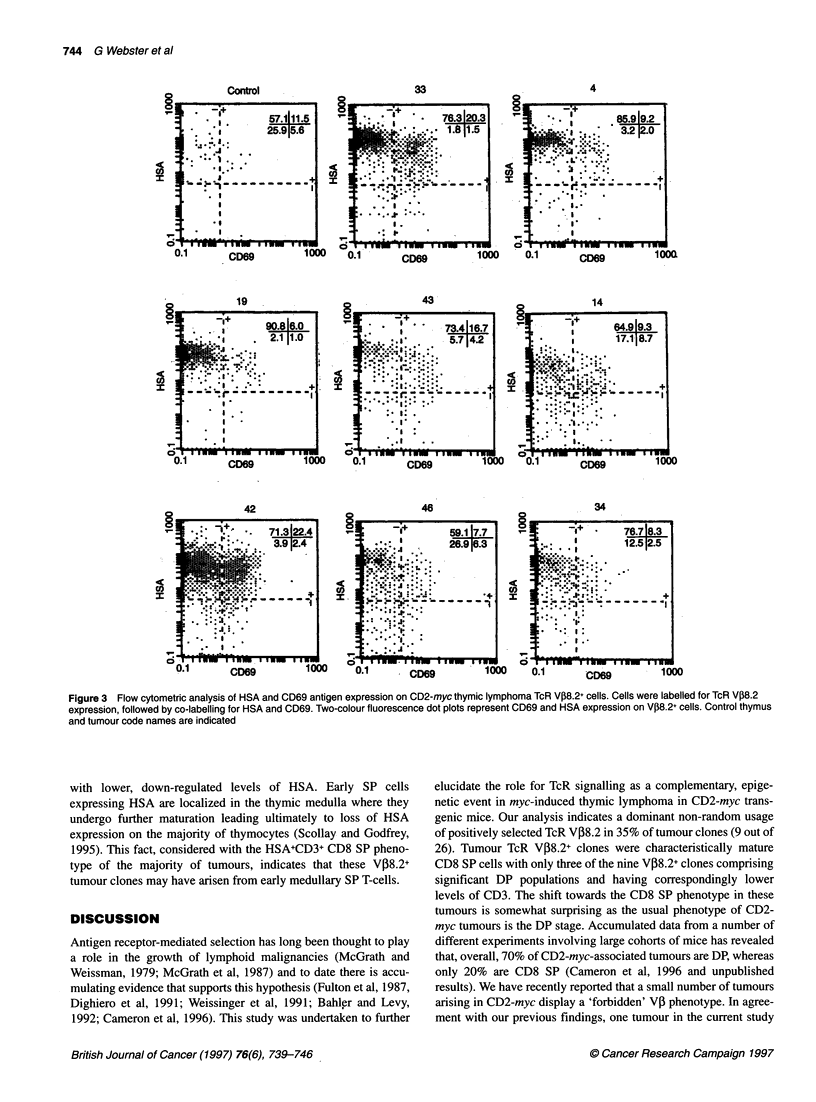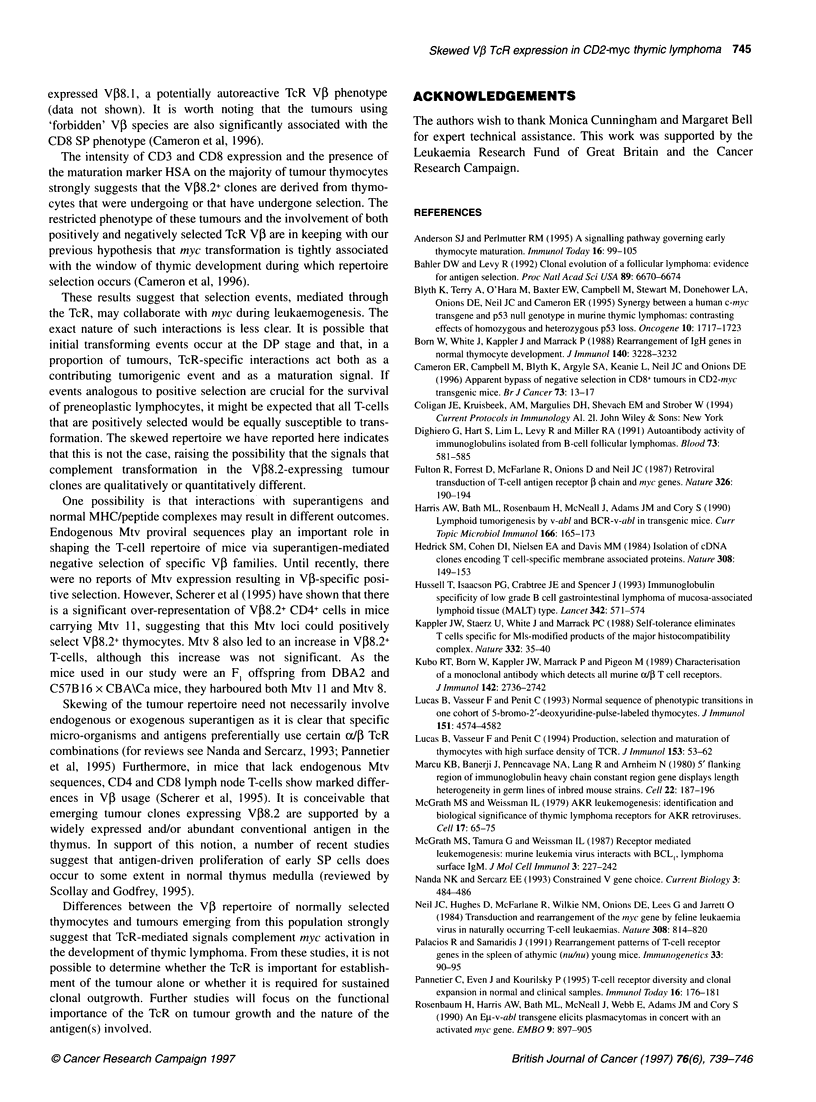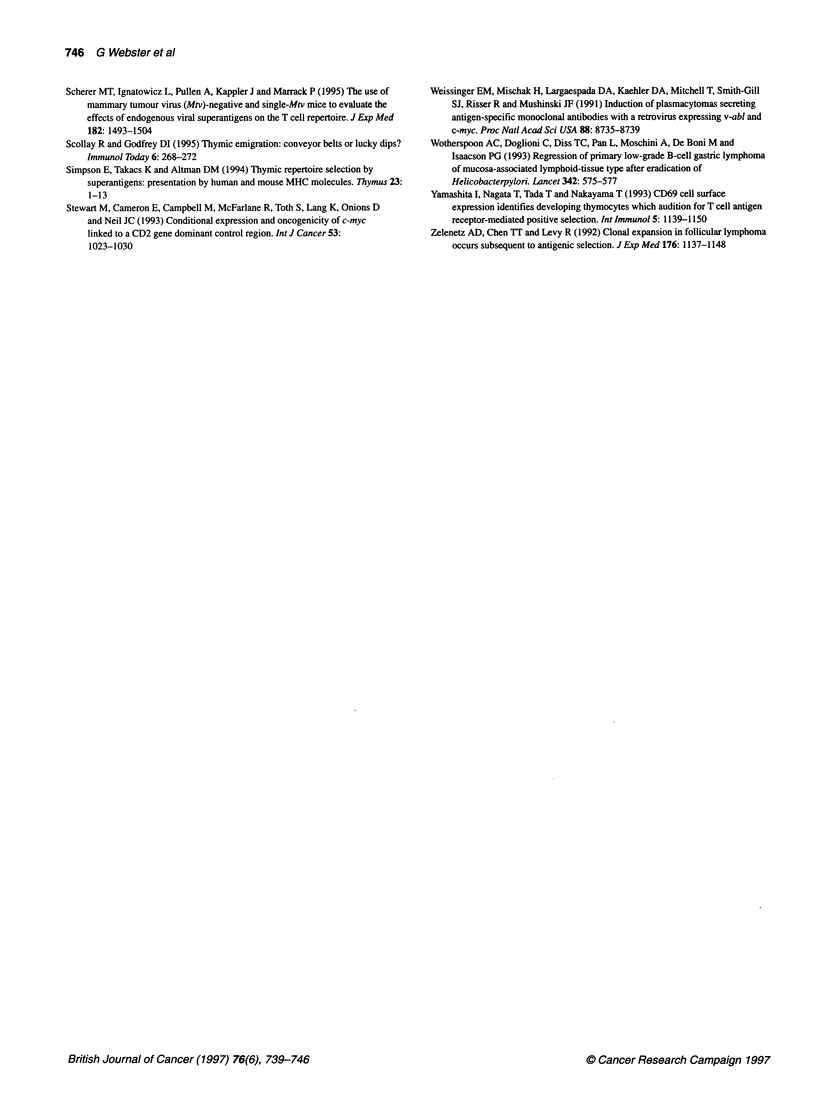# Skewed T-cell receptor Vbeta8.2 expression in transgenic CD2-myc induced thymic lymphoma: a role for antigen stimulation in tumour development?

**DOI:** 10.1038/bjc.1997.455

**Published:** 1997

**Authors:** G. Webster, D. E. Onions, J. C. Neil, E. R. Cameron

**Affiliations:** Department of Veterinary Pathology, University of Glasgow Veterinary School, UK.

## Abstract

**Images:**


British Journal of Cancer (1997) 76(6), 739–746
© 1997 Cancer Research Campaign

### Skewed T-cell receptor Vβ8.2 expression in transgenic CD2-*myc* induced thymic lymphoma: a role for antigen stimulation in tumour development?

**G Webster[1]*, DE Onions[1], JC Neil[1] and ER Cameron[2]**

[1]Department of Veterinary Pathology, University of Glasgow Veterinary School, Bearsden Road, Glasgow G61 1QH, UK; [2]Department of Veterinary Clinical Studies, University of Glasgow Veterinary School, Bearsden Road, Glasgow G61 1QH, UK

**Summary** Transgenic mice expressing the *c-myc* proto-oncogene under the control of the CD2-dominant control region show stochastic development of mainly clonal thymic lymphoma with long latency, indicating that cooperative events are needed for the development of the fully malignant phenotype. Previous studies have suggested that T-cell receptor-associated signals can contribute to tumour development. We have therefore used this transgenic model of T-cell transformation to determine whether antigen-specific responses could constitute an epigenetic event in lymphomagenesis. The T-cell receptor (TcR) repertoires of lymphoma clones were analysed with a panel of monoclonal antibodies (Abs) recognizing TcR Vβ chains. The Vβ repertoire of tumour clones arising in these mice was non-random with over-representation of Vβ8.2 TcR species. The majority of Vβ8.2$^+$ clones were of a mature CD3$^+$ CD8 single-positive (SP) phenotype. The biased TcR usage, together with a mature cell phenotype is consistent with the hypothesis that TcR-mediated signals cooperate with activated *myc* during T-cell transformation.

**Keywords:** CD2-*myc*; thymic lymphoma; Vβ gene usage; positive selection; endogenous antigen

A series of events, both genetic and epigenetic, are required to transform healthy cells to the fully malignant tumour phenotype. While many heritable genetic changes resulting in activation of cellular proto-oncogenes and/or inactivation of tumour-suppressor genes have been identified, the nature and spectrum of epigenetic events that can contribute to the transformation process is less well defined.

In malignancies of mature lymphoid cells it has been suspected that epigenetic events such as stimulation via conventional T- and B-cell antigen receptors may contribute to tumorigenesis. Some of the most convincing data have arisen from studies on human follicular lymphoma. Bahler and Levy (1992) reported that mutations of the immunoglobin heavy-chain variable region continued after follicular lymphoma development, indicating an important role for antigen in the clonal evolution of the lymphoma. Further, the non-random nature of mutations within the variable gene sequences of follicular lymphomas and the finding that surface immunoglobin expression is often preserved led Zelenetz et al (1992) to suggest that these lymphomas were subject to both positive and negative selection. The possibility that self antigens are playing a role in the selection process is supported by the finding that autoantibody production by follicular lymphomas is a relatively frequent occurrence (Dighiero et al, 1991).

The association between *H. lylori* infection and gastric B-cell lymphoma and the finding that antibiotic treatment can result in the regression of low-grade lymphomas (Wotherspoon et al, 1993) have resulted in the suggestion that antigen stimulation may play a role in the development of these lesions. Studies carried out by Hussell et al (1993) have indicated a role for T-cells in this chronic, possibly autoreactive, response. Similarly, the development of polyclonal plasmacytomas localized to the gut wall in Eμ-*myc*/*v-abl* double transgenic mice has led to the suggestion that tumour initiation involves stimulation by gut-associated antigen (Harris et al, 1990; Rosenbaum et al, 1990). In agreement with this hypothesis, antigenic stimulation has also been reported to contribute to the plasmacytomagenesis induced by a *myc*/*v-abl* retrovirus (Weissinger et al, 1991).

The possibility that signals mediated through the T-cell receptor (TcR) could contribute to the transformation of lymphoid cells arose from our studies on the pathogenesis of FeLV-induced thymic lymphomas. During analysis of proviral integrations in tumours, we observed one tumour with two separate retroviral transductions. One transducing virus contained the *myc* oncogene, while the other contained a processed and structurally intact TcR β-chain (Fulton et al, 1987). This observation suggested that oncogenic complementation between *myc* and the transduced TcR β-chain may have favoured clonal outgrowth, leading us to develop a murine model, the CD2-*myc* mice, to further investigate the involvement of the TcR in *myc*-induced lymphomagenesis (Cameron et al, 1996).

The CD2-*myc* transgenic mice develop thymic tumours exclusively. The long latency, the moderate incidence and the clonal nature of these tumours implies that in addition to *myc* deregulation, additional events are required for tumour development. Phenotypic examination of these tumours showed that they had either immature CD3$^+$CD4$^+$CD8$^+$ or mature CD3$^+$CD8 SP phenotypes. This limited phenotypic heterogeneity suggests that thymocytes are most susceptible to *myc* transformation at the

*Received 11 June 1996*
*Revised 24 January 1997*
*Accepted 13 March 1997*

*Correspondence to:* E Cameron

**Present address:* Wellcome Institute, Department of Biochemistry, University of Dundee, Dundee DD1 4HN, UK

developmental stage(s) when selection events are expected. We have previously reported that a small number of these tumours comprised T-cells that expressed a potentially autoreactive TcR Vβ chain (Cameron et al, 1996) and that such tumours were significantly associated with the mature CD3+CD8 SP phenotype. These results suggested that negative selection or clonal anergy may be bypassed and that endogenous antigen may generate additional proliferative signals.

To further define the nature and extent of TcR involvement in tumour induction/progression, we have placed the CD2-*myc* transgene on a genetic background in which a large number of TcR Vβ families are subjected to negative selection and examined the tumour T-cell repertoire. Surprisingly, there was an apparent bias in usage of non-deleted TcR Vβ8.2 in tumours that were also predominantly of a mature phenotype. Considered together, these data describe tumours comprising cells with characteristics of a mature and antigen-restricted phenotype, suggesting the TcR may mediate a signal(s) that can interact with *myc* in T-cell transformation.

### MATERIALS AND METHODS

### Transgenic mice

The generation of the CD2-*myc* transgenic mice has been described (Stewart et al, 1993). The *myc* transgenic mice (C57BL/6J and CBA/Ca mixture, hereafter referred to as B6/CBA) were backcrossed to DBA/2 mice. The resultant $F_1$ progeny were sacrificed when presenting with clinical signs of tumour development, and the thymic lymphomas were removed and frozen in liquid nitrogen as a single-cell suspension for flow cytometric analysis. Other tissues were also snap frozen for comparative analysis.

### Southern blot DNA analysis

Analysis of immunoglobin or T-cell receptor gene rearrangements were used to determine thymic lymphoma clonal complexity. High-molecular-weight DNA was prepared from thymic lymphocytes and intact kidney using the Nucleon II kit (Scotlab). Restriction enzyme digestion, agarose gel electrophoresis and DNA hybridization was performed as previously described (Neil et al, 1984). Digested DNA was transferred to Hybond N membranes (Amersham International) according to the manufacturer's protocol. Immunoglobulin gene rearrangements were detected using a 1.7-kb *Bam*HI/*Eco*RI fragment J11 from the Ig heavy-chain locus J cluster (Marcu et al, 1980). TcR gene rearrangements were detected using a 496-bp polymerase chain reaction (PCR)-generated fragment of the Cβ region derived from a 1.2-kb fragment of clone 86T5 (Hendrick et al, 1984) or a 750-bp *Eco*RI/*Hin*dIII fragment of Jβ2 from the TcR β J locus (Palacios and Samaridis, 1991). Probes were radiolabelled by random priming using [α-$^{32}$P]dCTP (3000 Ci mmol$^{-1}$, Amersham International) to specific activities of at least $1 \times 10^8$ c.p.m. per μg of DNA. Blots were washed to a final stringency of 0.1 × standard saline citrate (SSC); 0.5% sodium dodecyl sulphate (SDS) at 60°C.

### Monoclonal antibodies

The mouse IgG1 F23.2 (anti-Vβ 8.2) and mouse IgG2a F23.1 (anti-Vβ 8.1,.2,.3) Ab (Kappler et al, 1988) were obtained from tissue culture supernatants (hybridomas kindly provided by Dr P Marrack, Howard Hughes Medical Institute, Co, USA) and purified by protein A–Sepharose affinity chromatography. Biotinylation was performed using biotin-*N*-hydroxysuccinimide according to manufacturers recommendations (Amersham International). Biotinylated Ab recognizing TcR Vβ11, Vβ14 and Vβ8.1,.2 were purchased from Pharminigen. All biotinylated Abs were visualized with streptavidin-RED613 secondary reagent (Gibco BRL). Phenotypic characterization was performed using anti-CD4-PE, anti-CD8-FITC (Serotec, UK), anti-CD3ε-Quantum Red (Sigma), anti-CD69-FITC (Pharminigen) and anti-HSA-PE (Pharminigen). Total α/βTcR expression was determined using an Ab that reacts with all α/βTcR (Kubo et al, 1989) conjugated to Quantum Red (Sigma).

### Flow cytometric analysis of thymic lymphoma lymphocytes

Single-cell suspensions were recovered from liquid nitrogen into phosphate-buffered saline (PBS) containing 0.1% bovine serum albumin (BSA) and 0.2% sodium azide and were distributed in 96-well round-bottom microplates ($10^6$ cells per well). Cells were stained for 20 min at 4°C for each step in 100-μl volumes. For four-colour analysis, cells were incubated first with 1 μg of biotinylated mAbs recognizing TcR variable regions. The cells were washed and incubated with streptavidin-RED613 then sequentially exposed to optimal quantities of anti-CD3ε, anti-CD4 and anti-CD8 or to anti-pan TcRα/β, anti-HSA and anti-CD69. To account for the possibility of steric hindrance affecting Ab binding in four-colour analysis, single-stained controls were included for each tumour analysed. Thymocytes and splenic lymphocytes obtained from non-transgenic (B6/CBA × DBA/2)$F_1$ animals were used as controls. Cells were analysed on an Epics Elite cytometer (Coulter) using logarithmic scales for fluorescence data collection. Viable cells were gated by a combination of forward light scatter and 90° side scatter. A minimum of 10 000 tumour lymphocytes and 20 000 control thymocytes were analysed. The proportion of TcR Vβ8.3+ cells was indirectly determined by comparing binding of anti-Vβ8.1,.2,.3 (F23.1) and anti-Vβ8.1,.2 Ab. Similarly, the proportion of TcR Vβ8.1+ cells was determined by subtraction of Vβ8.3+ and Vβ8.2+ (F23.2) populations from the proportion of Vβ8.1,.2,.3+ cells (F23.1).

### RESULTS

### Biased usage of the Vβ8.2 T-cell antigen receptor in CD2 thymic lymphoma

The TcR repertoire in mice is strongly influenced by the products of endogenous mouse mammary tumour proviruses (MMTV; Mtv loci). Thymocytes expressing TcR Vβ elements that are reactive with MMTV-associated superantigens (SAg) undergo negative selection, resulting in deletion of the vast majority of cells; this is reflected by the almost complete absence of these cells from the peripheral T-cell population (Simpson et al, 1994). As (B6/CBA × DBA/2)$F_1$ mice carry numerous Mtv (Coligan et al, 1994), it was expected that a large number of TcR Vβ families would be deleted (e.g. Vβ3, 5, 6, 7, 8.1, 9, 11), while Vβ8.2, Vβ8.3 and Vβ14 would be among those spared (Coligan et al, 1994).

The Vβ repertoire of *myc*-induced T-cell lymphomas was investigated by screening a cohort of 20 tumours arising in CD2-*myc* transgenic mice on a B6/CBA × DBA/2 ($F_1$) background. We have previously reported that *myc*-induced thymic tumours can comprise cells displaying a 'forbidden' Vβ phenotype (Cameron

 © Cancer Research Campaign 1997

**Figure 1** Flow cytometric evaluation of TcR Vβ8.2 expression and DNA analyses of gene rearrangement in CD2-*myc* thymic lymphoma. The code name for each tumour is shown above. Corresponding kidney DNA (K) was analysed to indicate the position of the germline fragment (arrowed). *Eco*RI-or *Hin*dIII-digested DNA was transferred to nylon filters by Southern blotting procedures and hybridized with [α-$^{32}$P]dCTP-labelled J11 or Jβ2 probe, which detect Ig heavy-chain locus J cluster rearrangements or TcR β J locus rearrangements respectively. This analysis reveals clonal rearrangements with the loss of the germline fragment (arrowed) in some cases, indicating rearrangement of both alleles. For flow cytometric analysis, total tumour lymphocytes were labelled with a panel of anti-TcR Vβ Ab, as outlined in Materials and methods. Tumours expressing TcR Vβ8.2 are shown with corresponding DNA analysis. Markers indicating positive fluorescence regions (gate J) were set according to negative control Ab staining (gate F)

et al, 1996). In order to further investigate these results, we examined these 20 tumours with monoclonal antibodies for T-cells deleted on this background (Vβ8.1 and Vβ11) as well as T-cells for non-deleting Vβ species (Vβ8.2, Vβ8.3 and Vβ14). One tumour clone consisted of TcR Vβ8.1$^{+}$ cells, confirming our previous observation that tumours bearing potentially autoreactive T-cell receptors could arise in this system (data not included). However, an unexpected finding was the high proportion of tumours (8 out of 20) that contained substantial numbers of cells expressing Vβ8.2. No Vβ11$^{+}$, Vβ8.3$^{+}$ or Vβ14$^{+}$ tumour populations were detected and the remaining 11 tumours were uncharacterized with the panel of TcR Vβ Ab used in this study.

© Cancer Research Campaign 1997

Figure 2 CD3, CD4 and CD8 surface antigen co-expression on TcR Vβ8.2+ cells and TcR α/β expression on whole tumour lymphocytes. The code name for each tumour is shown. Cells were labelled for TcR Vβ8.2 expression followed by CD3, CD4 and CD8 co-labelling. Cells expressing Vβ8.2 were gated and subsequent CD3, CD4 and CD8 expression analysed on these cells. Because of steric hindrance of TcR α/β Ab binding in the presence of Vβ8.2 Ab, TcR α/β expression was determined for the whole tumour cell population. The regions indicating the positively labelled cells were set in relation to isotype-matched irrelevant negative controls labelled with the appropriate fluorochrome for each tumour. Regions indicated on CD3 and α/βTcR histograms refer to high and low levels of antigen density, which were determined using control thymocytes. Values for the percentage of positive cells are indicated

 © Cancer Research Campaign 1997

Previous studies on the CD2-*myc* transgenic mice have shown that tumours arising in these mice are usually clonal or oligoclonal. To investigate the clonal complexity of the thymic tumours, rearrangements of both the IgH locus and the TcR β gene were determined (Figure 1 and results not shown). We have found that non-productive IgH rearrangements, which are relatively frequent (30–50%) in normal T-cell development (Born et al, 1988), are useful markers of clonal identity in T-cell tumours (Blyth et al, 1995). As shown in Figure 1, rearrangement of one or both alleles of the immunoglobulin heavy-chain gene was a frequent occurrence. In tumours in which IgH rearrangements were not present, rearrangements of the Jβ2 or Cβ1 locus were analysed (Figure 1). As with previous results, this analysis revealed that CD2-*myc* lymphomas were largely clonal and showed that the tissues sampled were almost entirely composed of transformed cells. Six of the eight Vβ8.2$^+$ tumours (4, 14, 19, 34, 43, 46) appeared to represent single clones as defined by IgH or Jβ2 rearrangement patterns. Further IgH rearrangement patterns of tumours 33 and 42 suggest that these tumours were biclonal. This analysis was in general supported by the Vβ8.2$^+$ expression profiles as determined by flow cytometry (Figure 1). The Vβ8.2$^+$ expression profiles of tumours 4, 14 and 19 are represented as single peaks in agreement with the IgH Southern blot results. Tumour 42 is clearly biclonal as defined by IgH rearrangements but shows a single strongly positive peak for Vβ8.2$^+$ expression, indicating that this tumour consists of two Vβ8.2$^+$ clones. The other biclonal tumour (33) shows both Vβ8.2-positive and -negative clones. Although clonal by IgH rearrangements, tumours 34, 43 and 46 are more heterogeneous in their Vβ8.2 expression patterns. The reason for the disparity between the apparent clonality, as defined by IgH rearrangements, and the increased heterogeneity of Vβ8.2 expression is not clear. It is conceivable, however, that cell surface expression was downregulated on a proportion of the transformed thymocytes, perhaps because sustained expression was not required for the late stages of tumour growth.

In summary, this analysis indicates that the eight tumours were composed of ten distinct clones of which nine could be classified as Vβ8.2 positive. Analysis of IgH and Jβ2 rearrangements in the remaining 12 Vβ8.2-negative tumours revealed that these tumours were composed of a total of 16 clones. Overall, therefore, the 20 tumours in the cohort were composed of 26 clones, of which one expressed Vβ8.1 and nine expressed Vβ8.2 (35%). As it was possible that the high frequency of TcR Vβ8.2$^+$ tumour clones was simply a reflection of Vβ8.2 usage in normal untransformed T-cell populations, we examined the representation of Vβ8.2$^+$ cells in both TcR α/β$^+$ thymic populations and in Thy1.2$^+$ splenic populations from non-transgenic (DBA/2 × B6/CBA)$F_1$ controls. In adult thymus and spleen, Vβ8.2$^+$ cells represented 16.5 ± 2.9% ($n$ = 6) of α/β TcR$^+$ and 12.1 ± 1.5% ($n$ = 5) of Thy1.2$^+$ populations respectively. These data are of similar values to those obtained by others (Kappler et al, 1988). When compared with the tumour cohort in which 35% of clones expressed TcR Vβ8.2, these data suggest that the tumour Vβ repertoire was limited with an obvious skew towards TcR Vβ8.2 usage.

### TcR Vβ8.2$^+$ populations in CD2-myc thymic lymphoma resemble cells that have undergone or are undergoing selection

CD3, CD4 and CD8 co-expression was determined on gated Vβ8.2$^+$ clones to assess the developmental stage of these cells (Figure 2). The majority (six out of nine) of Vβ8.2$^+$ clones comprised cells of a CD4-CD8$^+$ phenotype with a high level of CD3 expression (termed CD3$^+$CD8 SP), suggesting that these clones arose from cells that had completed positive selection. TcR expression of the tumours was also determined using flow cytometry, however double labelling with Vβ8.2 and α/β TcR Ab resulted in reduced staining compared with single-stained positive controls; as a result this analysis was carried out on whole tumour populations. Although of lower intensity, TcR expression patterns generally paralleled those of CD3 with CD3$^+$CD8 SP tumours 33, 4, 19 and 14 showing distinct single peaks of intermediate level (Figure 2). As with the pattern of Vβ8.2 expression in tumour 43, CD3 and TcR levels are more heterogeneous in this tumour. The CD3 expression pattern of tumour 42 reflects the biclonal nature of this tumour and suggests that one of the Vβ8.2$^+$ clones is CD3hi CD8$^+$ while the other Vβ8.2$^+$ clone has lower levels of CD3. Analysis of TcR expression in this tumour also indicates that there are two separate clones that differ in TcR intensity.

TcR Vβ8.2$^+$ clones in tumours 46 and 34 contained significant immature DP populations, which expressed correspondingly lower levels of CD3 than seen in other Vβ8.2$^+$ tumour populations (Figure 2). By contrast, the clones that did not express Vβ elements recognized by the mAb panel were more heterogeneous with respect to CD3, CD4 and CD8 expression (data not shown). Fourteen of these clones were examined by flow cytometry; five were composed primarily of CD3$^+$CD8 SP cells while seven had significant DP populations with varying levels of CD3 expression. Of the remainder, one clone was of a predominant CD3$^-$CD8$^+$ SP phenotype and the other showed major subsets in both the CD3$^+$CD8 SP and CD3$^+$DP compartments.

In addition to mature T-cell populations, CD8 SP cells are also represented in the thymus as a transitional pre-double-positive (DP) population. Such cells do not express detectable CD3 or TcRα chain, and CD8 antigen expression is defined as low compared with antigen density on mature T-cells (Lucas et al, 1993; Anderson and Perlmutter, 1995). As six of the nine Vβ8.2$^+$ clones show high levels of CD3 and CD8 expression levels together with intermediate levels of TcR, they appear to be representative of a post-selection population rather than the pre-double-positive population of CD3$^-$CD8$^{lo}$ cells. TcR Vβ8.2$^+$ clones in tumours 46 and 34, and one of the two clones in tumour 42, expressed low levels of α/β TcR, which was in agreement with the predominant CD3$^{lo}$ DP immature phenotype.

While phenotypic analysis of tumours for CD3, CD4 and CD8 was indicative of a mature, post-selection T-cell phenotype, we sought to define further the developmental compartment of these cells using the conventional activation and maturation markers CD69 and HSA (heat stable antigen) respectively. CD69 is an activation marker that is rapidly induced on mature peripheral T-cells after stimulation through the TcR (Yamashita et al, 1993). CD69 has also been shown to be developmentally regulated in its expression on thymocytes. In particular, it is believed that CD69 identifies a subpopulation of thymocytes that are, or have recently been, stimulated through their T-cell receptor complexes during positive and negative selection (Yamashita et al, 1993). HSA characterizes immature thymocytes from DN to early SP (Lucas et al, 1993, 1994). As shown in Figure 3, all Vβ8.2$^+$ tumour clones exhibited CD69 and HSA characteristics similar to those seen in Vβ8.2$^+$ cells undergoing normal thymocyte development in control mice. For each clone, the majority of Vβ8.2$^+$ cells were of a HSA$^+$ CD69$^-$ phenotype, with a small fraction of cells expressing CD69

© Cancer Research Campaign 1997

**Figure 3** Flow cytometric analysis of HSA and CD69 antigen expression on CD2-*myc* thymic lymphoma TcR Vβ8.2+ cells. Cells were labelled for TcR Vβ8.2 expression, followed by co-labelling for HSA and CD69. Two-colour fluorescence dot plots represent CD69 and HSA expression on Vβ8.2+ cells. Control thymus and tumour code names are indicated with lower, down-regulated levels of HSA. Early SP cells expressing HSA are localized in the thymic medulla where they undergo further maturation leading ultimately to loss of HSA expression on the majority of thymocytes (Scollay and Godfrey, 1995). This fact, considered with the HSA+CD3+ CD8 SP phenotype of the majority of tumours, indicates that these Vβ8.2+ tumour clones may have arisen from early medullary SP T-cells.

### DISCUSSION

Antigen receptor-mediated selection has long been thought to play a role in the growth of lymphoid malignancies (McGrath and Weissman, 1979; McGrath et al, 1987) and to date there is accumulating evidence that supports this hypothesis (Fulton et al, 1987, Dighiero et al, 1991; Weissinger et al, 1991; Bahler and Levy, 1992; Cameron et al, 1996). This study was undertaken to further elucidate the role for TcR signalling as a complementary, epigenetic event in *myc*-induced thymic lymphoma in CD2-*myc* transgenic mice. Our analysis indicates a dominant non-random usage of positively selected TcR Vβ8.2 in 35% of tumour clones (9 out of 26). Tumour TcR Vβ8.2+ clones were characteristically mature CD8 SP cells with only three of the nine Vβ8.2+ clones comprising significant DP populations and having correspondingly lower levels of CD3. The shift towards the CD8 SP phenotype in these tumours is somewhat surprising as the usual phenotype of CD2-*myc* tumours is the DP stage. Accumulated data from a number of different experiments involving large cohorts of mice has revealed that, overall, 70% of CD2-*myc*-associated tumours are DP, whereas only 20% are CD8 SP (Cameron et al, 1996 and unpublished results). We have recently reported that a small number of tumours arising in CD2-*myc* display a 'forbidden' Vβ phenotype. In agreement with our previous findings, one tumour in the current study

© Cancer Research Campaign 1997

expressed Vβ8.1, a potentially autoreactive TcR Vβ phenotype (data not shown). It is worth noting that the tumours using 'forbidden' Vβ species are also significantly associated with the CD8 SP phenotype (Cameron et al, 1996).

The intensity of CD3 and CD8 expression and the presence of the maturation marker HSA on the majority of tumour thymocytes strongly suggests that the Vβ8.2$^+$ clones are derived from thymocytes that were undergoing or that have undergone selection. The restricted phenotype of these tumours and the involvement of both positively and negatively selected TcR Vβ are in keeping with our previous hypothesis that *myc* transformation is tightly associated with the window of thymic development during which repertoire selection occurs (Cameron et al, 1996).

These results suggest that selection events, mediated through the TcR, may collaborate with *myc* during leukaemogenesis. The exact nature of such interactions is less clear. It is possible that initial transforming events occur at the DP stage and that, in a proportion of tumours, TcR-specific interactions act both as a contributing tumorigenic event and as a maturation signal. If events analogous to positive selection are crucial for the survival of preneoplastic lymphocytes, it might be expected that all T-cells that are positively selected would be equally susceptible to transformation. The skewed repertoire we have reported here indicates that this is not the case, raising the possibility that the signals that complement transformation in the Vβ8.2-expressing tumour clones are qualitatively or quantitatively different.

One possibility is that interactions with superantigens and normal MHC/peptide complexes may result in different outcomes. Endogenous Mtv proviral sequences play an important role in shaping the T-cell repertoire of mice via superantigen-mediated negative selection of specific Vβ families. Until recently, there were no reports of Mtv expression resulting in Vβ-specific positive selection. However, Scherer et al (1995) have shown that there is a significant over-representation of Vβ8.2$^+$ CD4$^+$ cells in mice carrying Mtv 11, suggesting that this Mtv loci could positively select Vβ8.2$^+$ thymocytes. Mtv 8 also led to an increase in Vβ8.2$^+$ T-cells, although this increase was not significant. As the mice used in our study were an $F_1$ offspring from DBA2 and C57B16 × CBA\Ca mice, they harboured both Mtv 11 and Mtv 8.

Skewing of the tumour repertoire need not necessarily involve endogenous or exogenous superantigen as it is clear that specific micro-organisms and antigens preferentially use certain α/β TcR combinations (for reviews see Nanda and Sercarz, 1993; Pannetier et al, 1995) Furthermore, in mice that lack endogenous Mtv sequences, CD4 and CD8 lymph node T-cells show marked differences in Vβ usage (Scherer et al, 1995). It is conceivable that emerging tumour clones expressing Vβ8.2 are supported by a widely expressed and/or abundant conventional antigen in the thymus. In support of this notion, a number of recent studies suggest that antigen-driven proliferation of early SP cells does occur to some extent in normal thymus medulla (reviewed by Scollay and Godfrey, 1995).

Differences between the Vβ repertoire of normally selected thymocytes and tumours emerging from this population strongly suggest that TcR-mediated signals complement *myc* activation in the development of thymic lymphoma. From these studies, it is not possible to determine whether the TcR is important for establishment of the tumour alone or whether it is required for sustained clonal outgrowth. Further studies will focus on the functional importance of the TcR on tumour growth and the nature of the antigen(s) involved.

### ACKNOWLEDGEMENTS

The authors wish to thank Monica Cunningham and Margaret Bell for expert technical assistance. This work was supported by the Leukaemia Research Fund of Great Britain and the Cancer Research Campaign.

### REFERENCES

Anderson SJ and Perlmutter RM (1995) A signalling pathway governing early thymocyte maturation. *Immunol Today* **16**: 99–105

Bahler DW and Levy R (1992) Clonal evolution of a follicular lymphoma: evidence for antigen selection. *Proc Natl Acad Sci USA* **89**: 6670–6674

Blyth K, Terry A, O'Hara M, Baxter EW, Campbell M, Stewart M, Donehower LA, Onions DE, Neil JC and Cameron ER (1995) Synergy between a human c-*myc* transgene and p53 null genotype in murine thymic lymphomas: contrasting effects of homozygous and heterozygous p53 loss. *Oncogene* **10**: 1717–1723

Born W, White J, Kappler J and Marrack P (1988) Rearrangement of IgH genes in normal thymocyte development. *J Immunol* **140**: 3228–3232

Cameron ER, Campbell M, Blyth K, Argyle SA, Keanie L, Neil JC and Onions DE (1996) Apparent bypass of negative selection in CD8$^+$ tumours in CD2-*myc* transgenic mice. *Br J Cancer* **73**: 13–17

Coligan JE, Kruisbeek, AM, Margulies DH, Shevach EM and Strober W (1994) *Current Protocols in Immunology* Al. 21. John Wiley & Sons: New York

Dighiero G, Hart S, Lim L, Levy R and Miller RA (1991) Autoantibody activity of immunoglobulins isolated from B-cell follicular lymphomas. *Blood* **73**: 581–585

Fulton R, Forrest D, McFarlane R, Onions D and Neil JC (1987) Retroviral transduction of T-cell antigen receptor β chain and *myc* genes. *Nature* **326**: 190–194

Harris AW, Bath ML, Rosenbaum H, McNeall J, Adams JM and Cory S (1990) Lymphoid tumorigenesis by v-*abl* and BCR-v-*abl* in transgenic mice. *Curr Topic Microbiol Immunol* **166**: 165–173

Hedrick SM, Cohen DI, Nielsen EA and Davis MM (1984) Isolation of cDNA clones encoding T cell-specific membrane associated proteins. *Nature* **308**: 149–153

Hussell T, Isaacson PG, Crabtree JE and Spencer J (1993) Immunoglobulin specificity of low grade B cell gastrointestinal lymphoma of mucosa-associated lymphoid tissue (MALT) type. *Lancet* **342**: 571–574

Kappler JW, Staerz U, White J and Marrack PC (1988) Self-tolerance eliminates T cells specific for Mls-modified products of the major histocompatibility complex. *Nature* **332**: 35–40

Kubo RT, Born W, Kappler JW, Marrack P and Pigeon M (1989) Characterisation of a monoclonal antibody which detects all murine α/β T cell receptors. *J Immunol* **142**: 2736–2742

Lucas B, Vasseur F and Penit C (1993) Normal sequence of phenotypic transitions in one cohort of 5-bromo-2′-deoxyuridine-pulse-labeled thymocytes. *J Immunol* **151**: 4574–4582

Lucas B, Vasseur F and Penit C (1994) Production, selection and maturation of thymocytes with high surface density of TCR. *J Immunol* **153**: 53–62

Marcu KB, Banerji J, Penncavage NA, Lang R and Arnheim N (1980) 5′ flanking region of immunoglobulin heavy chain constant region gene displays length heterogeneity in germ lines of inbred mouse strains. *Cell* **22**: 187–196

McGrath MS and Weissman IL (1979) AKR leukemogenesis: identification and biological significance of thymic lymphoma receptors for AKR retroviruses. *Cell* **17**: 65–75

McGrath MS, Tamura G and Weissman IL (1987) Receptor mediated leukemogenesis: murine leukemia virus interacts with $BCL_1$, lymphoma surface IgM. *J Mol Cell Immunol* **3**: 227–242

Nanda NK and Sercarz EE (1993) Constrained V gene choice. *Current Biology* **3**: 484–486

Neil JC, Hughes D, McFarlane R, Wilkie NM, Onions DE, Lees G and Jarrett O (1984) Transduction and rearrangement of the *myc* gene by feline leukaemia virus in naturally occurring T-cell leukaemias. *Nature* **308**: 814–820

Palacios R and Samaridis J (1991) Rearrangement patterns of T-cell receptor genes in the spleen of athymic (*nu/nu*) young mice. *Immunogenetics* **33**: 90–95

Pannetier C, Even J and Kourilsky P (1995) T-cell receptor diversity and clonal expansion in normal and clinical samples. *Immunol Today* **16**: 176–181

Rosenbaum H, Harris AW, Bath ML, McNeall J, Webb E, Adams JM and Cory S (1990) An Eμ-v-*abl* transgene elicits plasmacytomas in concert with an activated *myc* gene. *EMBO* **9**: 897–905

© Cancer Research Campaign 1997 

Scherer MT, Ignatowicz L, Pullen A, Kappler J and Marrack P (1995) The use of mammary tumour virus (*Mtv*)-negative and single-*Mtv* mice to evaluate the effects of endogenous viral superantigens on the T cell repertoire. *J Exp Med* **182**: 1493–1504

Scollay R and Godfrey DI (1995) Thymic emigration: conveyor belts or lucky dips? *Immunol Today* **6**: 268–272

Simpson E, Takacs K and Altman DM (1994) Thymic repertoire selection by superantigens: presentation by human and mouse MHC molecules. *Thymus* **23**: 1–13

Stewart M, Cameron E, Campbell M, McFarlane R, Toth S, Lang K, Onions D and Neil JC (1993) Conditional expression and oncogenicity of *c-myc* linked to a CD2 gene dominant control region. *Int J Cancer* **53**: 1023–1030

Weissinger EM, Mischak H, Largaespada DA, Kaehler DA, Mitchell T, Smith-Gill SJ, Risser R and Mushinski JF (1991) Induction of plasmacytomas secreting antigen-specific monoclonal antibodies with a retrovirus expressing v-*abl* and c-*myc*. *Proc Natl Acad Sci USA* **88**: 8735–8739

Wotherspoon AC, Doglioni C, Diss TC, Pan L, Moschini A, De Boni M and Isaacson PG (1993) Regression of primary low-grade B-cell gastric lymphoma of mucosa-associated lymphoid-tissue type after eradication of *Helicobacterpylori*. *Lancet* **342**: 575–577

Yamashita I, Nagata T, Tada T and Nakayama T (1993) CD69 cell surface expression identifies developing thymocytes which audition for T cell antigen receptor-mediated positive selection. *Int Immunol* **5**: 1139–1150

Zelenetz AD, Chen TT and Levy R (1992) Clonal expansion in follicular lymphoma occurs subsequent to antigenic selection. *J Exp Med* **176**: 1137–1148

*© Cancer Research Campaign 1997*